# Neuroprotective Potential of *Acmella oleracea* Aerial Parts and Root Extracts: The Role of Phenols and Alkylamides Against Neuropathic Pain

**DOI:** 10.3390/nu17162588

**Published:** 2025-08-08

**Authors:** Valentina Ferrara, Beatrice Zonfrillo, Maria Bellumori, Marzia Innocenti, Laura Micheli, Valentina Maggini, Daniel Venturi, Eugenia Gallo, Patrizia Bogani, Lorenzo Di Cesare Mannelli, Carla Ghelardini, Nadia Mulinacci, Fabio Firenzuoli

**Affiliations:** 1 Department of Neuroscience, Psychology, Drug Research and Child Health (NEUROFARBA), Section of Pharmacology and Toxicology, University of Florence, 50139 Florence, Italy; valentina.ferrara@unifi.it (V.F.); daniel.venturi1@unifi.it (D.V.); lorenzo.mannelli@unifi.it (L.D.C.M.); carla.ghelardini@unifi.it (C.G.); 2 Department of Neuroscience, Psychology, Drug Research and Child Health (NEUROFARBA), Section of Pharmaceutical and Nutraceutical Sciences, University of Florence, via Ugo Schiff 6, 50019 Sesto Fiorentino, Italy; beatrice.zonfrillo@unifi.it (B.Z.); maria.bellumori@unifi.it (M.B.); marzia.innocenti@unifi.it (M.I.); nadia.mulinacci@unifi.it (N.M.); 3 CERFIT, Research and Innovation Center in Phytotherapy and Integrated Medicine, Careggi University Hospital, 50134 Florence, Italyeugenia.gallo@unifi.it (E.G.); fabio.firenzuoli@unifi.it (F.F.); 4 Department of Biology, University of Florence, 50019 Sesto Fiorentino, Italy; patrizia.bogani@unifi.it

**Keywords:** spilanthol, alkylamides, phenolic compounds, neuroprotection, neuropathic pain, oxaliplatin

## Abstract

***Background***: Chemotherapy-induced neuropathic pain is a major side effect of antineoplastic treatment. This study investigated the neuroprotective potential of *Acmella oleracea* L. extracts containing the N-alkylamide spilanthol, phenolic acids, and glycosylated flavonoids. ***Methods***: Hydroalcoholic extracts of aerial parts (AP) and roots (R) of in vitro seedlings were quali-quantitatively characterized by HPLC-DAD-MS and by ^1^H-NMR. Different concentrations (15–150 µg/mL) of AP and R were tested in SH-SY5Y cells differentiated into neurons exposed to oxaliplatin (10 µM), assessing cell viability (MTT), cytotoxicity (LDH), SOD activity, and expression of ATF-3, Ire1α, and Nf-H genes. To evaluate the impact on neuropathic pain, CD-1 mice were treated intraperitoneally with oxaliplatin (2.4 mg/kg), the effect of AP and R extracts (200–1200 mg/kg) were measured by the cold plate test. ***Results***: AP extract was rich in phenols and alkylamides, whereas R extract showed higher phenolic levels but lower alkylamides content. Both extracts significantly reduced mortality and cytotoxicity and counteracted oxidative imbalance by enhancing SOD activity. Gene expression analysis confirmed their neuroprotective effects. In vivo, oxaliplatin induced a 50% reduction in pain threshold, while acute treatment with AP and R extracts dose-dependently alleviated neuropathic pain. Despite the lower spilanthol content in R extract, its efficacy was comparable to AP, suggesting an important role of phenolic compounds. ***Conclusions***: Extracts from both aerial parts and roots of *A. oleracea* show promise in alleviating chemotherapy-induced neuropathy through mechanisms not solely related to spilanthol. Further studies to elucidate the contribution of phenolic components are desirable.

## 1. Introduction

Oxaliplatin-induced peripheral neurotoxicity (OIPN) is a severe limiting side effect in the management of patients undergoing chemotherapy who are using this anticancer drug [1]. Although oxaliplatin-induced peripheral neuropathy is not a life-threatening condition, it has a substantial impact on patients’ quality of life. It is often considered highly debilitating, as it can not only lead to dose reductions or discontinuation of chemotherapy, but also represent a major source of chronic pain in cancer survivors [2]. The nervous damage induces neuropathic hypersensitivity characterized by hyperalgesia, paresthesia, allodynia, tingling, and shooting pain [3].

Despite the abundance of available drugs [4], OIPN remains inadequately alleviated by conventional analgesics [5], given that its molecular mechanisms and pathogenesis are not yet fully understood. Consequently, research is progressively directing its attention towards innovative therapeutic alternatives, including the exploration of bioactive compounds found in medicinal plants [6]. The terrestrial environment serves as a huge source, housing approximately 300,000 plant species, with only 15% of them having undergone a study of their biological effects. Notably, among various habitats, the Amazon rainforest stands out as one of the most prolific natural product reservoirs [7].

In this regard, *Acmella oleracea* (L.) R.K. Jansen (Asteraceae), commonly known as jambù, is a medicinal plant native to Brazil. It is also known as the toothache plant due to its traditional use in the treatment of oral pain. The phytochemical composition of the plant has been extensively investigated by several authors. The essential oil from the inflorescences of *Acmella oleracea*, resulting in a rich content of monoterpene and sesquiterpene hydrocarbons, has shown antimicrobial and insecticidal activities [8]. To date, the plant is best known for the molecule spilanthol, an alkylamide formed by a condensation reaction between a fatty acid and a decarboxylated amino acid, which is mainly responsible for the biological effects attributed to *Acmella oleracea*. Spilanthol is found in the aerial parts of the plant, especially in the flowers, and its concentration in the different extracts depends on the type of cultivation and the extraction method [9]. The plant also contains flavonoids and other phenolic compound previously identified in aerial parts of plants grown by conventional and hydroponic methods [10] and in *in vitro* seedlings [11]. The roots contain a lower amount of alkylamides and approximately twice the total phenols in respect to the aerial parts [11]. Minor secondary metabolites such as triterpenoids and phytosterols have also been reported [12]. Phenolic compounds have been associated with the antioxidant properties of the plant [13], but their possible role in enhancing or not enhancing the effects of spilanthol has not been well studied so far. Thus, the aim of the study was to evaluate the in vitro and in vivo effect of two *Acmella oleracea* extracts, obtained from the aerial parts and roots of in vitro seedlings, mitigating the neurotoxic effects of oxaliplatin.

The extracts were chemically characterized to determine the different concentration of total phenols, total alkylamides, and spilanthol. SH-SY5Y, a human neuroblastoma cell line differentiated in neurons [14,15], was used for an initial comprehensive assessment of cellular and molecular responses to the neurotoxicity evoked by oxaliplatin. Subsequently, further assessments were performed in vivo on mice developing painful chemotherapy-induced neuropathy [16].

## 2. Materials and Methods

### 2.1. Samples of Acmella Oleracea

Aerial parts and roots of *Acmella oleracea* were obtained from in vitro seedlings. Five different organogenesis-derived regenerating lines were produced according to Maggini and colleagues [17]. Extractions were performed on representative samples of the two tissues obtained by combining the five regenerating lines which showed similar content of secondary metabolites [11]. Hydroalcoholic extracts were prepared following the procedure reported in [11]. Briefly, a hydroalcoholic extraction (80% *v*/*v*) with ultrasounds (10 min, 60 °C) was carried out on dried leaves and roots (1:20 *w*/*v*) in coarse powder, followed by a liquid/liquid extraction 1:2 *v/v* with hexane to remove some of the lipophilic molecules and chlorophylls. To obtain an easy-to-handle powder for the oral administration in the animal test, the defatted hydroalcoholic extracts of aerial parts and roots were combined with maltodextrin (1:1 *w*/*w*) and lyophilized to give AP and R samples. The quantitative data were expressed on the dried weight of AP and R samples.

### 2.2. Chemical and Reagents

Maltodextrin (dextrose equivalent 4.0–6.0), D_2_O, and CDCl_3_, and all the other reagents and solvents of High-Performance Liquid Chromatography (HPLC) grade or analytical grade were purchased from Sigma-Aldrich (Steinheim, Germany). Ultrapure water for Mass Spectrometry (MS) analyses was from Milli-Q-system (Millipore, Molsheim, France). The standard sample of spilanthol was kindly provided by Indena S.p.A (Milan, Italy); the purity grade was of 47.64% *w*/*w*, evaluated by HPLC at 229 nm. The pure standards used for quantitation of phenolic acids and flavonoids were chlorogenic acid (purity ≥ 95%) and quercitrin (purity ≥ 97%), both purchased from Sigma Aldrich (Steinheim, Germany).

### 2.3. HPLC-DAD-ESI-MS Analyses

The dried extracts were analyzed by HPLC-DAD-ESI-MS (Agilent Technologies, Palo Alto, CA, USA) according to [11]. Briefly, the instrument was an HP 1260 Infinity II liquid chromatograph equipped with a Diode Array Detector (DAD) detector and MS detector all from Agilent Technologies (Palo Alto, CA, USA). The column was a RaptorTM ARC-18 of 150 × 3 mm, 5 µm (Restek, Milan, Italy), and flow rate of 0.4 mL min^−1^. Solvent A was water at pH 3.2 by formic acid, and solvent B acetonitrile; A varied from 5 to 25% in 5 min, from 25 to 50% in 20 min, from 50 to 100% in 5 min, with a final plateau of 5 min; total time of analysis 37 min.

A five-point calibration curve at λ 229 nm of spilanthol (purity grade by HPLC 47.6% *w*/*w*) was applied for spilanthol and alkylamides; the mother solution was 238 µg/mL, a linearity range of 0–3.57 μg and R^2^ 0.9985. A five-point calibration line of chlorogenic acid (0.458 mg/mL) at λ 330 nm (linearity range 0–3.66 µg, R^2^ 0.9998) was used for the phenolic acids. Flavonoids were determined using a five-point calibration curve of quercitrin (quercetin-3 L rhamnoside) evaluated at λ 350 nm (linearity range 0–2.45 µg; R^2^ 0.9999).

Spilanthol was quantified by MS with a five-point calibration curve; mother solution 23.8 μg/mL, linearity range 0–0.0014 μg, R^2^ 0.9956. The applied method was previously validated [11].

Flow injection analyses were conducted to optimize electrospray ionization (ESI) parameters for the generation of the spilanthol molecular ion [M + H]^+^ 222.2 m/z. The acquisition was performed in a Selected Ion Monitoring (SIM) scan, positive ionization mode with fragmentor 120 V, and the following parameters: nitrogen flow rate 10.5 mL min^−1^, drying gas temperature 300 °C, nebulizer pressure 1035 Torr, capillary voltage 3500 V. Polyphenols and alkylamides were identified by comparison with authentic pure standards, based on retention times, UV spectra, and mass spectral data; compounds without available standards were tentatively identified through retention times and characteristic MS fragmentation patterns, supported by comparison with the literature data [10,11].

### 2.4. ^1^H-NMR Analyses

Proton spectra were carried out on a Bruker instrument Advance 400 MHz (Bruker, Bremen, Germany). AP and R dried extracts were analyzed in D_2_O and in CDCl_3_ (50 mg/mL, while spilanthol was dissolved in CDCl_3_ (12 mg/mL); AP and R samples in both the deuterated solvents were only partially dissolved (saturated solutions). The acquisition of proton spectra was carried out by applying Bruker sequence “zg” with temperature 298 K; relaxation delay 12 s; pulse width 7 μs; and number of scans 12.

### 2.5. SH-SY5Y Cell Culture Protocol and Differentiation

SY-SY5Y cultures were set up according to the previously reported method, with minor modifications [15]. Human neuroblastoma SH-SY5Y cell line was maintained in DMEM High Glucose/Ham’s F12 Mixture Medium (1:1) (Merck, Milan, Italy), supplemented with 10% FBS (Euroclone S.p.A., Milan, Italy), 2 mM l-Glutamine (Merck, Milan, Italy), 100 U/mL penicillin (Merck, Milan, Italy), 100 μg/mL streptomycin (Merck, Milan, Italy), at 37 °C in 5% CO_2_ in a cell culture incubator. Once cells reached 70–80% confluence, cells were harvested and seeded into a multi-well plates at the desired density.

For differentiation, SH-SY5Y cells were pre-treated with Retinoic Acid (RA) 10 µM (Merck, Milan, Italy) in DMEM/Nutrient Mixture F-12 Ham (F12) 1% FBS. The medium was changed every 2 days for 6 days. Starting from day 6, the medium was changed to Neurobasal-A (NB) medium (Life Technologies, Rockford, IL, USA) supplemented with brain-derived neurotrophic factor (BDNF) 100 ng/mL (Sino Biological, Inc., Beijing, China), N2 supplement (1X) (Life Technologies, Rockford, IL, USA), and Amphotericin B (ATB, 2.5 µg/mL) (Merck, Milan, Italy) for 2 additional days. After the differentiation, cells were incubated with *Acmella oleracea* AP and R extracts (15, 50, and 150 µg/mL), alone and in combination with oxaliplatin (10 µM).

### 2.6. Pharmacological Treatments

Following differentiation, SH-SY5Y cells were incubated with oxaliplatin at a concentration of 10µM in Neurobasal medium for 24 h. SH-SY5Y cells were treated with hydroalcoholic extracts of *Acmella oleracea* from aerial parts (AP) and roots (R) at concentrations of 15–50–150 µg/mL. The extracts were obtained by solubilizing 50 mg/mL lyophilized samples in dimethyl sulfoxide (DMSO, Merck, Milan, Italy); the final dilution of DMSO was 1%.

### 2.7. Cell Viability Assay

Cell viability was assessed using the reduction of 3-(4,5-dimethylthiazol-2-yl)-2,5-diphenyltetrazolium bromide (MTT; Merck, Milan, Italy), which reflects the functional activity of the mitochondrial compartment. SH-SY5Y cells were plated at a density of 3 × 10^4^ cells/well in 96-well plates (Corning, Tewksbury, MA, USA) and after differentiation, cells were incubated with oxaliplatin (10 µM) and *Acmella oleracea* extracts (R and AP) (15–150 µg/mL), for 24 h. At the end of the treatment, MTT was added to the medium at a final concentration of 1 mg/mL and then the colored formazan crystals were dissolved in 200 µL of dimethyl sulfoxide (DMSO, Merck, Milan, Italy). Absorbance was measured at 550 nm. Data are presented as mean ± S.E.M. of N = 3 independent experiments, each performed with six technical replicates

### 2.8. LDH Release Assay

Cytotoxicity was evaluated through the release of lactate dehydrogenase (LDH) in the culture medium of differentiated SH-SY5Y cell line.

SH-SY5Y cells were plated at a density of 1.5 × 10^4^ cells/well in 24-well plates (Corning, Tewksbury, MA, USA), and after differentiation, cells were incubated with oxaliplatin (10 µM) and R HAE or AP HAE (15–150 µg/mL) for 24 h.

Following the treatments, the release of LDH was determined using the Cytotoxicity Detection Kit (Roche 11644793001, Roche Diagnostics GmbH Roche Applied Science Mannheim, Germany) following the manufacturer’s instructions. Protein concentration was quantified using the BCA assay (Merck, Milan, Italy). The amount of LDH released in each sample was normalized to protein concentration. Data are presented as mean ± S.E.M. of N = 3 independent experiments, each per-formed with six technical replicates

### 2.9. Superoxide Dismutase (SOD) Assay

Oxidative stress was evaluated by performing an SOD assay on differentiated SH-SY5Y. SH-SY5Y cells were plated at a density of 1.5 × 10^4^ cells/well in 24-well plates (Corning, Tewksbury, MA, USA), and after differentiation, cells were incubated with oxaliplatin (10 µM) and AP or R (15–150 µg/mL) for 24 h. SOD activity was measured in the supernatant using the SOD Assay Kit (Merck, Milan, Italy), following the manufacturer’s instructions. Protein concentration was quantified using the bicinchoninic acid (BCA) assay (Merck, Milan, Italy). SOD activity in each sample was normalized to protein concentration. Data are presented as mean ± S.E.M. of N = 3 independent experiments, each per-formed with six technical replicates.

### 2.10. RNA Isolation, Reverse Transcription, and Real-Time Polymerase Chain Reaction (RT-PCR)

Total RNA was isolated from SH-SY5Y cell lines (5 × 10^5^ cells/well in 6-well plate; Corning, Tewksbury, MA, USA). In total, 250 nanograms of RNA for SH-SY5Y were retrotranscribed using the PrimeScript^TM^ RT reagent Kit with gDNA eraser (Takara Bio, Kusatsu, Japan). RT-PCR was performed using SsoAdvanced Universal SYBR^®^ Green Supermix (Bio-Rad, Hercules, CA, USA) following the thermal profile suggested by the kit. For SH-SY5Y, the following validated human primers for hERN1, hATF3, hNEFH, hACTB, and hGAPDH (qHsaCID0016338, qHsaCED0002279, qHsaCID0011672, qHsaCED0036269, and qHsaCED0038674) were purchased from Bio-Rad. The differential expression of transcripts was normalized on the expression level of housekeeping genes. Data are presented as mean ± S.E.M. of N = 3 independent experiments, each performed with six technical replicates

### 2.11. Animals

CD-1 adult male mice (20–25 g) used in the experiments were provided from Envigo RMS SRL (Varese, Italy). The animals were fed a standard laboratory diet and tap water ad libitum and housed in a room kept at 23 ± 1 °C with a 12 h light/dark cycle (light on at 7 a.m.). Environmental enrichment was provided to all animals throughout the study to promote natural behaviors and improve welfare. This included nesting materials, shelters, and objects for manipulation, which were regularly refreshed to maintain interest and stimulation. Animals were randomly assigned to the different experimental groups to reduce the risk of systematic bias. Group allocation was performed using a computer-generated randomization procedure (Microsoft Excel) to assign a random number to each subject by applying the =RAND() function. The list of animals was then sorted in ascending order based on these random values, and mice were allocated sequentially to the different experimental groups. Each experimental group consisted of eight animals (N = 8). All animal manipulations were carried out according to the Directive 2010/63/EU of the European Parliament and of the European Union council (22 September 2010) on the protection of animals used for scientific purposes and conformed to the International Association for the Study of Pain (IASP) guidelines on ethical standards for the investigation of experimental pain in animals. Given the objective of our study, to evaluate novel approaches for chemotherapy-induced neuropathic pain, analgesics were not administered, as they would interfere with the validity of the pain model and confound experimental outcomes. Although the presence of pain is inherent to the model, animals were monitored daily to assess their general health and well-being. Specific attention was paid to signs such as reduced activity or social interaction, pallor (indicative of anemia), grooming behavior, body condition, and progressive weight loss. Humane endpoints were predefined to ensure animal welfare. Criteria included severe and sustained weight loss (>20%), signs of systemic distress (e.g., dehydration, persistent lethargy, or lack of responsiveness), or other indicators of deteriorating general health. However, none of the animals enrolled in the study reached these predefined endpoints, and therefore, no animals were euthanized for humane reasons during the experimental timeline. The ethical policy of the University of Florence complies with the Guide for the Care and Use of Laboratory Animals of the US National Institutes of Health (NIH Publication No. 85–23, revised 1996; University of Florence assurance number: A5278-01). We performed an *a priori* power analysis using G*Power (version 3.1.9.2) to determine the appropriate sample size. The calculation was based on pilot data, assuming an effect size of 1.6, a significance level (α) of 0.05, and a statistical power (1–β) of 0.8.

As a result, a minimum of 8 animals per group was determined to be sufficient to detect statistically meaningful differences.

### 2.12. Oxaliplatin-Induced Neuropathic Pain Model and Pharmacological Treatments

Oxaliplatin (2.4 mg/kg) was dissolved in 5% glucose solution and intraperitoneally (i.p.) administered for 5 consecutive days every week for two weeks (10 injections) [18,19] (with minor modifications).

Control animals received an equivalent volume of 5% glucose saline solution intraperitoneally administered.

Regarding animal treatments with plant-derived products, hydroalcoholic extracts of *Acmella oleracea* from roots and aerial parts, R and AP (200, 600, and 1200 mg/kg), were dissolved in 1% carboxymethylcellulose sodium salt (CMC; Sigma-Aldrich, Milan, Italy) and administered *per os*; spilanthol (2.38 and 7.146 mg/kg) was dissolved in saline/DMSO/Tween20 (90%, 5%, 5%) and administered subcutaneously; all treatments were tested by acute administration when neuropathy was well established (day 14) [20]. Control groups received vehicles.

### 2.13. Assessment of Thermal Allodynia (Cold Plate Test)

To minimize interaction between the operator and the animals, each mouse was gently removed from its home cage and placed on the surface of a cold plate apparatus (Ugo Basile, Varese, Italy) maintained at a constant temperature of 4 ±  1 °C. Animal movement was restricted using an open-top Plexiglas cylindrical chamber (10 cm in diameter, 15 cm in height). Pain-related behaviors (i.e., lifting and licking of the hind paw) were observed and the time (s) of the first sign was recorded. The cut-off for paw licking or licking was set at 30 s [20].

### 2.14. Statistical Analysis

Chemical data were expressed as mean ± SD. One-way ANOVA followed by Tukey’s post hoc test was used to assess significant differences between AP and R. Statistical analysis was performed in RStudio (2024.09.0+375).

Biological results were expressed as mean ± S.E.M. and analysis of variance (one-way ANOVA) was performed. A Bonferroni significant difference procedure was used as post hoc comparison. Data were analyzed using the “Origin 9.1” software (OriginLab, Northampton, MA, USA). *p*-values less than 0.05 were considered significant; * *p* < 0.05; ** *p* < 0.01; *** *p* < 0.001 vs. control, and ^ *p* < 0.05; ^^ *p* < 0.01; ^^^ *p* < 0.001 vs. treatment. To reduce experimental bias, all in vivo procedures were carried out by researchers blinded to the treatment assignments.

## 3. Results

### 3.1. Chemical Characterization of the Extracts

**Figure 1:**
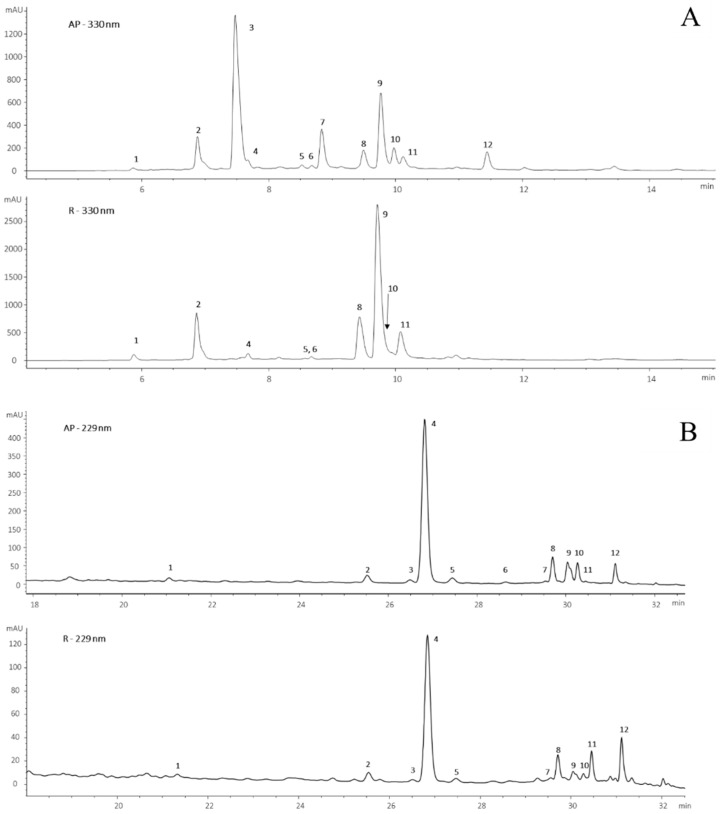
(**A**) Chromatographic profiles at 330 nm of the hydroalcoholic extracts of aerial parts (AP) and roots (R). (1) 3-caffeoylquinic acid; (2) 5-caffeoylquinic acid; (3) Caffeoylmalic acid; (4) Caffeic acid; (5) Rutin; (6) Miquelianin; (7) feruloylmalic acid; (8) 3,4-di-O-caffeoylquinic acid; (9) 3,5-di-O-caffeoylquinic acid; (10) 4,5-di-O-caffeoylquinic acid; (11) Rosmarinic acid; (12) Petasiphenol. (**B**) Chromatographic profile at 229 nm of the hydroalcoholic extracts of aerial parts (AP) and roots (R). (1) N-isobutyl-2-nonene-6,8-dynamide; (2) (2E)-N-isobutylundeca-2-ene-8,10-dynamide; (3) Spilanthol isomer; (4) Spilanthol: (2E,6Z,8E)-N-isobutyl-2,6,8-decatrienamide; (5) Spilanthol isomer; (6) (2E)-N-(2-methylbutyl)-2-undecene-8,10-dynamide; (7) (2E,7Z)-N-(isobutyl)-2,7-tridecadiene-10,12-diynamide; (8) Homospilanthol: (2E,6Z,8E)-N-(2-methylbutyl)-deca-2,6,8-trienamide; (9) unknown; (10) (2E,7Z)-N-isobutyl-2,7-decadienamide; (11) (2E,4E,8Z,10Z)-N-isobutyldodeca-2,4,8,10-tetraenamide; (12) Decen-2-oic acid isobutylamide.

The extracts from roots and aerial parts were analyzed by HPLC-DAD-MS, showing the chromatographic profiles of Figure 1. AP and R extracts detected at 330 nm (Figure 1A) presented phenolic compounds belonging to the classes of cinnamoyl derivatives and flavonoids, while the same extracts detected at 229 nm showed the presence of alkylamides (Figure 1B) according to our previous data [11]. Two independent numberings have been applied for the identification of the compounds in the two extracts.

The extracts from the two tissues showed quantitative differences in the two main classes of secondary metabolites. The aerial parts contained a lower content in total phenols with respect to the total alkylamides, with a concentration of spilanthol of 2.2 mg/g DE. In contrast, the roots contained almost double the total phenols (14.15 mg/g DE), but negligible amounts of alkylamides and spilanthol (Table 1). Depending on the part of the plant used, the extraction procedure allowed us to obtain two dry extracts with very different relative ratios between phenolic compounds and alkylamides, allowing us to evaluate the effects of these different combinations in cell and animal models.

As for the chemical characterization of the dry extracts, spilanthol was quantified by HPLC-DAD at the maximum absorption wavelength of 229 nm, while the quantification by HPLC-MS was applied to control possible co-elution with other molecules. The two calibration curves built by DAD and by MS lead to very similar concentration values (Table 1). The little higher amount obtained for AP extract with HPLC-DAD (10% more) is attributable to the coelution of interfering molecules absorbing at 229 nm, acceptable when analyzing complex extracts. Since the DAD detector is more accessible a less expensive, it was preferred to evaluate the concentrations of spilanthol in the AP and R samples.

**Figure 2:**
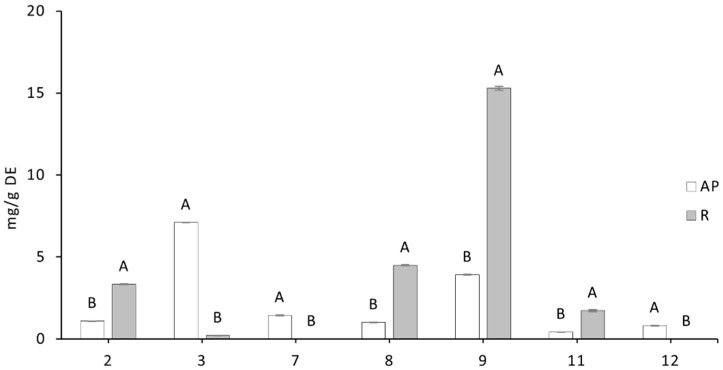
Distribution of the main phenolic compounds (approx. 90% of the total content) in the two dried extracts from aerial parts (AP) and roots (R). (2) 5-caffeoylquinic acid; (3) caffeoylmalic acid; (7) feruloylmalic acid; (8) 3,4-di-caffeoylquinic acid; (9), 3,5-di-caffeoylquinic acid; (11) rosmarinic acid; (12), petasiphenol (numbering according to Figure 1). Data are expressed as a means of triplicates (N = 3) ± SD. Statistically significant differences between AP and R were evaluated by one-way ANOVA followed by Tukey’s post hoc test and are indicated with different letters (*p* < 0.0001).

Although the chromatographic profiles at 330 nm were qualitatively similar in aerial parts and roots, and the analytes identified were mostly represented by caffeoyl derivatives (Figure 1A), significant quantitative differences were found in the relative abundance of phenols (Figure 2). Compound 3 (feruloyl malic acid) is the most abundant in aerial parts, followed by 3,5-caffeoylquinic acid (9). This latter phenol is the major component in roots, accounting for more than 50% of the total phenolic content. Caffeoylmalic acid (2), 1,4-di-caffeoylmalic acid (8) and rosmarinic acid (11) are present in both tissues whereas compounds 7 (feruloylmalic acid) and 12 (petasiphenol) are found only in the aerial parts. The di-caffeoylmalic acid isomers were identified by comparing their retention times and mass spectra with those of a previously characterized coffee sample [21]. Overall, the two AP and R extracts contain a pool of structurally very similar phenols, but with significant quantitative differences in all the components of the samples.

To further investigate the composition of the two extracts, some experiments by ^1^H-NMR spectroscopy were also performed. This technique enables the detection of different types of protons in organic compounds coexisting within a complex sample, based on their chemical shifts (ppm values) [22]. The intense signals at 3.2–4.2 ppm in the spectra of AP and R in D_2_O confirmed the presence of quinic acid units and glycoside moieties, as well as the anomeric protons close to 5.3 ppm (Figure S1) [23]. For the two samples, the signals at 6.5–7.5 ppm, related to the aromatic protons of the main phenolic compounds (listed in Figure 2), were almost overlapped, confirming the presence of a very similar pattern of phenolic structures in both extracts. The CDCl_3_ spectra of the two extracts, compared with the reference spectrum of spilanthol, confirmed the higher content of alkylamides in the AP sample. The ^1^H-NMR data agreed with the results highlighted by the HPLC-DAD-MS analyses (Table 1 and Figure 1).

### 3.2. Evaluation of the Neuroprotective Activity of Acmella Oleracea Extracts in an In Vitro Model of Oxaliplatin-Induced Neurotoxicity

**Figure 3:**
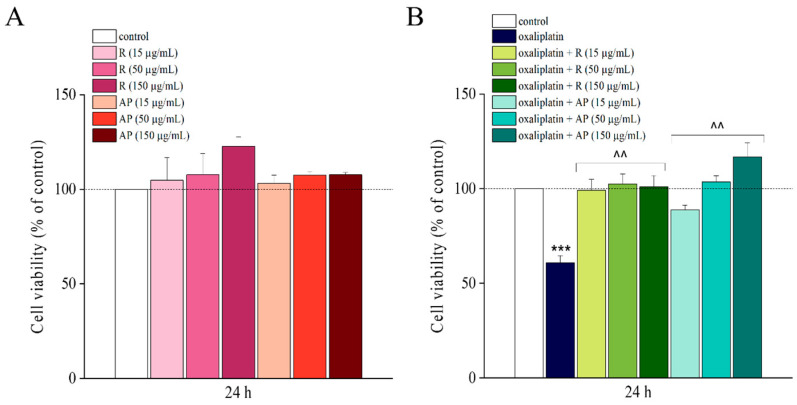
Cell viability analysis, MTT assay. Cell viability was assessed in the SH-SY5Y cell line following a 24 h treatment with *Acmella oleracea* extracts R and AP at three distinct concentrations (15–150 μg/mL) alone (**A**) and in combination with oxaliplatin (10 μM) (**B**) using the MTT assay. All results are presented as a percentage relative to the control group, which was arbitrarily set at 100%. Results are expressed as the mean ± SEM of three independent experiments (N = 3), each performed with six technical replicates per condition. Statistical analysis was carried out using a one-way ANOVA, followed by a Bonferroni post hoc comparison using Origin 9.1 software. *** *p* < 0.001 vs. control; ^^ *p* < 0.01 vs. oxaliplatin.

The safety of *Acmella oleracea* extracts, R and AP (15, 50, 150 µg/mL) was verified by MTT assay on SH-SY5Y cells after 24 h of treatment (Figure 3A). Based on these results, the same concentrations were used to study the potential protective effects against oxaliplatin toxicity. Oxaliplatin 10 µM (Figure 3B) induced a viability decrease by about 40%. Both extracts significantly prevented oxaliplatin-induced cell mortality at all the concentrations tested. AP acted with a concentration-dependent trend while R showed the same neuroprotection activity for all concentrations.

**Figure 4:**
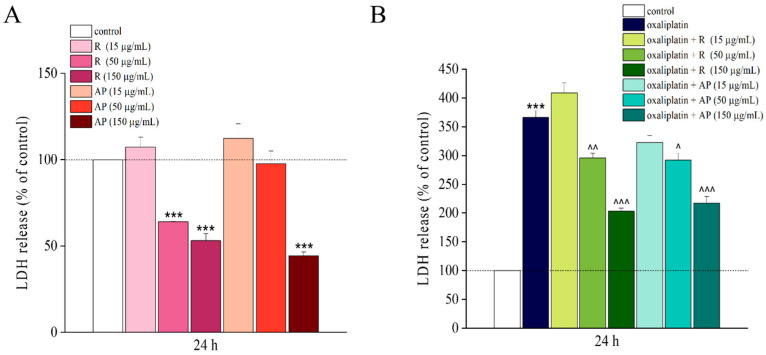
Cell cytotoxicity analysis, LDH assay. Cell cytotoxicity was evaluated in the SH-SY5Y cell line following a 24 h treatment with *Acmella oleracea* extracts, R and AP at three distinct concentrations (15, 50, and 150 μg/mL) alone (**A**) and in combination with oxaliplatin (10 μM) (**B**) using the lactate dehydrogenase (LDH) assay. All results are presented as a percentage relative to the control group, which was arbitrarily set at 100%. Results are expressed as the mean ± SEM of three independent experiments (N = 3), each performed with six technical replicates per condition. Protein concentration was quantified by bicinchoninic acid (BCA) assay. Statistical analysis was carried out using a one-way ANOVA, followed by a Bonferroni post hoc comparison. *** *p* < 0.001 vs. control; ^ *p* < 0.05, ^^ *p* < 0.01 and ^^^ *p* < 0.001; and vs. oxaliplatin. The data were analyzed using Origin 9.1 software.

Afterwards, cellular toxicity was evaluated by using the LDH (lactate dehydrogenase) assay, in the presence of R or AP extracts alone and in combination with oxaliplatin for 24 h. Highest concentrations of R and AP reduced LDH release *per se* and significantly prevented the LDH release (in comparison to control) evoked by oxaliplatin (Figure 4B). Both extracts acted in a concentration-dependent manner (Figure 4B).

### 3.3. Effect of Acmella Oleracea Extracts on SOD Activity

**Figure 5:**
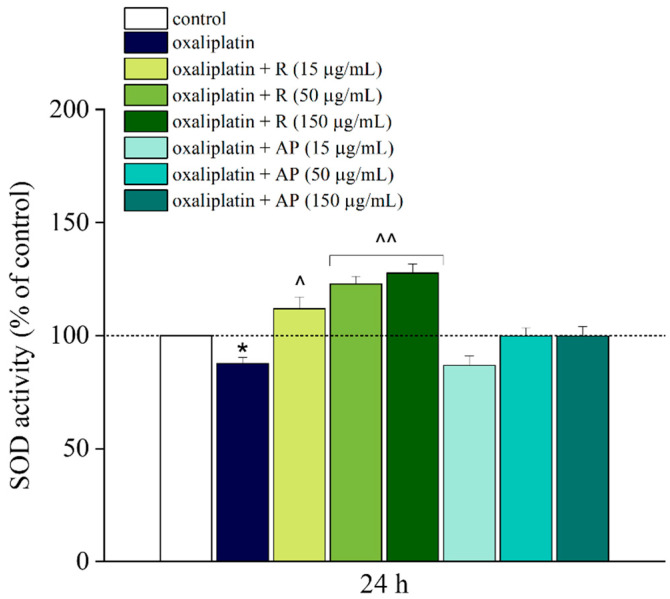
Oxidative stress measurement, SOD activity. Oxidative imbalance was evaluated by performing SOD activity assay on SH-SY5Y cell line following a 24 h treatment with *Acmella oleracea* extracts (R and AP) at three distinct concentrations (15, 50 and 150 μg/mL) in combination with oxaliplatin (10 μM). All results are presented as a percentage relative to the control group, which was arbitrarily set at 100%. Results are expressed as the mean ± SEM of three independent experiments (N = 3), each performed with six technical replicates per condition. Protein concentration was quantified by bicinchoninic acid (BCA) assay. Statistical analysis was carried out using a one-way ANOVA, followed by a Bonferroni post hoc comparison. * *p* < 0.05 vs. control; ^ *p* < 0.05 and ^^ *p* < 0.01 vs. oxaliplatin. The data were analyzed using Origin 9.1 software.

R and AP extracts were tested on SH-SY5Y cells treated with oxaliplatin (10 µM) which evoked an oxidative imbalance, slightly reducing SOD activity after 24 h of treatment. All concentrations of R, in combination with oxaliplatin, led to an increase in levels of superoxide dismutase compared to oxaliplatin alone (Figure 5).

### 3.4. Analysis of Gene Expression of ATF3, Ire1α, and Nf-H

**Figure 6:**
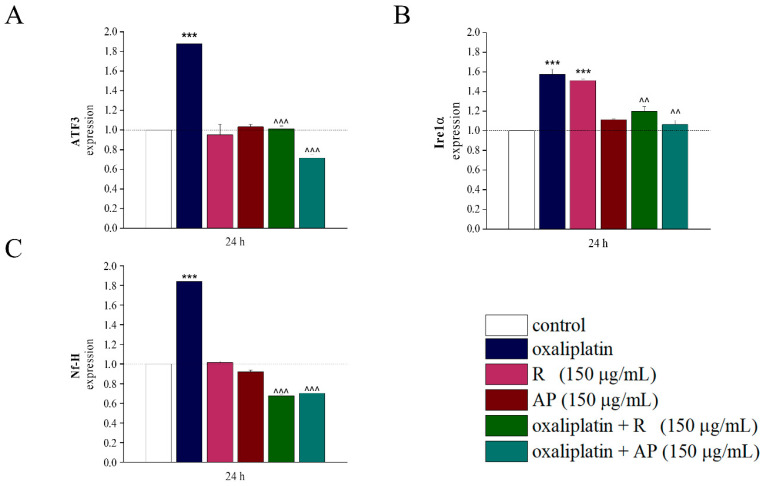
Gene expression analysis. RT-PCR of SH-SY5Y cell line treated for 24 h with *Acmella oleracea* extracts (R and AP) (150 μg/mL) alone and in combination with oxaliplatin (10 μM). The control was arbitrarily set at 1 and the mRNA levels normalized with the expression of GAPDH and β2 microglobulin, chosen as housekeeping genes. (**A**) ATF3, (**B**) Ire1α and (**C**) Nf-H genes were analyzed. Results are expressed as the mean ± SEM of three independent experiments (N = 3), each performed with six technical replicates per condition. *** *p* < 0.001 vs. control; ^^ *p* < 0.01 and ^^^ *p* < 0.001 vs. oxaliplatin.

ATF3 (Activating Transcription Factor 3), Ire1α (Inositol-Requiring Enzyme 1 Alpha), known for their involvement in cellular stress [24], and Nf-H (neurofilament heavy chain), recognized as a marker of neurotoxicity compared to the control [25], were studied in SH-SY5Y cells. R and AP (150 µg/mL, 24 h) did not modify gene expression of ATF3 and Nf-H. Only AP (150 µg/mL) led to a significant increase in expression of Irea1α gene. Oxaliplatin (10 µM, 24 h) significantly increased all the analyzed genes. Both the extracts prevented these alterations. The differential expression of transcripts was normalized to the expression level of the housekeeping genes GAPDH and β2 microglobulin, genes that are not influenced by the treatments (Figure 6).

### 3.5. R and AP Extracts Decrease Oxaliplatin-Induced Neuropathic Pain in Mice

Neuropathy was induced in mice by intraperitoneal daily administration of oxaliplatin (2.4 mg/kg) for 5 consecutive days each week for 2 weeks [19]. On day 14, when neuropathy was well established, each extract was acutely *per os* administered in the dose range 200–1200 mg/kg. Table 2 shows the absolute amount of total phenols, spilanthol, and total alkylamides in the administered doses of AP and R samples calculated from the concentrations (expressed in mg phenols/g DE) reported in Table 1.

**Figure 7:**
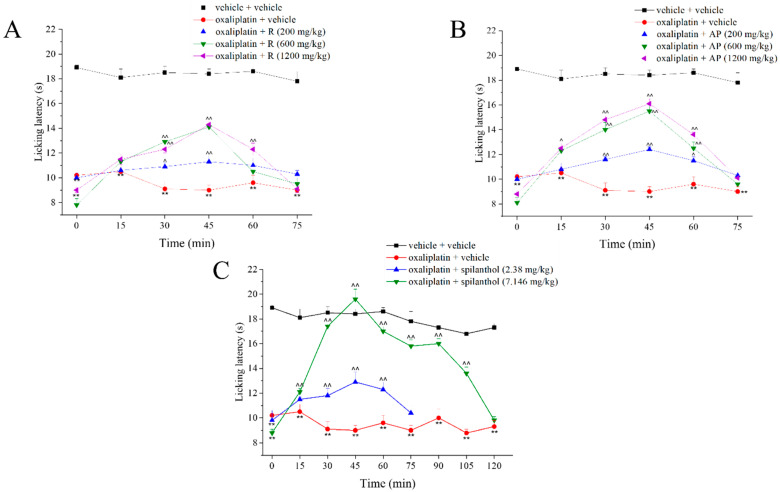
Pain threshold evaluation. Effect of R (**A**), AP (**B**), and spilanthol (**C**) on oxaliplatin-treated mice. Oxaliplatin (2.4 mg/kg) was administered for 5 consecutive days every week for 2 weeks. R, AP (200, 600 and 1200 mg/kg, p.o.) and spilanthol (2.38 and 7.14 mg/kg, s.c.) were administered acutely when neuropathy was established (day 14). Thermal allodynia was measured by cold plate test 15 min after the administration. The data shown represent the mean ± SEM of 8 mice per experimental group. Trained observers were not informed about the specific treatment of each animal group the tests were carried out on. ** *p* < 0.01 vs. vehicle + vehicle; ^ *p* < 0.05 and ^^ *p* < 0.01 vs. oxaliplatin + vehicle.

The pain threshold to a thermal stimulus was evaluated every 15 min until the end of the effect by using the cold plate test (Figure 7A,B). R and AP dose-dependently increased the pain threshold 15, 30, 45, and 60 min after administration, with the effect ending at 75 min. AP showed a slightly better profile.

The effect of spilanthol standard was also evaluated at two different doses and the subcutaneous administration was chosen to favor the absorption of this compound. The lower dose (2.38 mg/kg) is comparable to the amount contained in the highest dose of AP (2.64 mg/kg, Table 2). As shown in Figure 7C, spilanthol 2.38 mg/kg is significantly active from 30 to 60 min after administration, noting that this effect is lower compared to that induced by AP 1200 mg/kg. A three-fold higher dose of spilanthol was required to obtain complete reversal of oxaliplatin-induced neuropathic pain.

## 4. Discussion

Oxaliplatin-induced neuropathy is an important dose-limiting toxic effect boosting the research of alternative chemotherapeutic treatments. Our previous work showed the therapeutic potential of *Echinacea purpurea* extracts (i.e., an *n*-hexane extract rich in alkylamides and a butanolic extract rich in polyphenols) in neuropathic pain treatment [20]; thus, we investigated another medicinal plant, *Acmella oleracea*, rich in the same secondary metabolites [11]. To prevent low yield and high variability of active constituents, in vitro seedling cultures were obtained by a standardized cultivation method [17].

The extracts of *Acmella oleracea* selected for this study had different contents of secondary metabolites. The extract from the aerial part contained mainly phenolic compounds and about one third alkylamides, whereas the root extract was richer in phenols with negligible amounts of alkylamides like spilanthol. The different ratios between the two classes of metabolites in the extracts allowed for an investigation of the possible different activity of the different parts of the plant. This distinct phytochemical profile allowed us to examine the combined or individual effects of these two classes of compounds on neuroprotective mechanisms.

The biological data highlighted the anti-neuropathic properties of the plant. In particular, the two main phytochemical components, alkylamides and phenols, were active. In neuronal cells, both AP, the extract rich in spilanthol and phenols, and R, the extract with ten times lower spilanthol and twice the phenols, showed a similar protective effect against oxaliplatin-induced neurotoxicity. These extracts, depending on concentration, prevented the decrease in cell viability as evidenced by the MTT assay and the drug-induced cytotoxicity evaluated by LDH release.

The fact that both extracts exhibited similar neuroprotective effects, despite differences in spilanthol and phenol concentrations, suggests that phenolic compounds may play a central role in mediating the protective effect against neurotoxicity.

Additionally, an improvement in redox balance was observed. Treatment with oxaliplatin slightly reduced the activity of SOD, a crucial enzyme for protection against oxidative stress. Notably, platinum-based drugs cause an alteration of mitochondrial function followed by the disruption of the respiratory chain function and an increased production of reactive oxygen species (ROS) [26]. The root extract R, rich in phenolic compounds, reversed this trend, significantly enhancing SOD activity across all tested concentrations. This result highlights the importance of the antioxidant properties of phenolic compounds in counteracting oxaliplatin-induced oxidative stress, a mechanism known to underline the neurotoxicity associated with this chemotherapy [3]. The antioxidant properties of phenolic compounds, such as caffeoylquinic acids and ferulic acid derivatives, are well documented, and their potential in counteracting oxidative stress could explain part of the observed neuroprotective activity [13].

Interestingly, *Acmella oleracea* extracts act up to the transcriptional level normalizing the expression of genes altered by oxaliplatin. The analysis of gene expression for markers associated with cellular stress (Ire1α was related to endoplasmic reticulum stress, [27]; ATF3 with intracellular oxidative stress, mitochondrial dysfunction, cell apoptosis, [28]) and neurotoxicity (Nf-H, [29]) provided further evidence supporting the neuroprotective effect of the extracts. Oxaliplatin induced a significant increase in the expression of these genes, as expected in cases of neurotoxic damage. However, both extracts exhibited a modulatory effect, preventing the drug-induced upregulation of gene expression. This result points out that *Acmella oleracea* acts not only at the cellular level but also at the transcriptional level, modulating key molecular pathways involved in the cellular stress response. This suggests that the neuroprotective activity of the extracts is not limited to the enhancement of redox potential but also involves gene regulation mechanisms associated with stress response and neuronal survival.

On this basis, the extracts were tested in vivo in a mice model of oxaliplatin-induced neuropathy. AP and R in the dose range 200–1200 mg/kg reduced neuropathic pain after a single injection in a dose-dependent manner. These data are in line with recent evidence in which *Acmella oleracea* extract has been demonstrated to possess anti-allodynic activity in a preclinical model of spared nerve injury induced neuropathic pain [30]. AP exhibited a more favorable trend in efficacy and duration (although the difference in effect was not statistically significant). These results suggest that the combination of spilanthol and phenols present in aerial parts and roots contributes to an additive anti-neuropathic effect. The response confirms the role of alkylamides (e.g., spilanthol) in pain relief but strongly highlights the relevance of phenols. In fact, R extract—despite containing a small amount of spilanthol (0.13 mg/kg in the dose of 600 mg DE/kg bwt)—exerts significant anti-neuropathic effects. This consideration is also supported by the finding that spilanthol standard, administered at a dose approximately twenty times higher (2.38 mg/kg), achieved a lower level of efficacy. This suggests that the pharmacological properties of extracts in vivo are not solely due to the alkylamides and spilanthol, but rather to the interaction between spilanthol and phenolic compounds, present in the dried extract. This point warrants further investigation to clarify the mechanism of action of the single phenols detected in root and aerial parts of *Acmella oleracea* to identify the specific compounds responsible for the observed analgesic effect. The identification and quantification of phenols in these extracts, in addition to spilanthol, have proven crucial for the possible future development of novel therapeutic strategies for the management of neuropathic pain with *Acmella oleracea* extracts.

## 5. Conclusions

This study highlights the neuroprotective and anti-neuropathic potential of *Acmella oleracea* extracts in oxaliplatin-induced toxicity models (both in vitro and in vivo). Both aerial part and root extracts, showed comparable efficacy in preserving cell viability, reducing oxidative stress, modulating stress-related gene expression, and alleviating pain. Although the root extract contained ten times less spilanthol, it was the richest in total phenolic suggesting a central role for phenolics, potentially acting synergistically with alkylamides.

However, some limitations must be acknowledged. The contribution of individual phenols remains unclear, and the mechanism of action has not yet been fully elucidated. Moreover, pharmacokinetic properties and long-term safety of the extracts were not assessed.

Future studies should focus on identifying the main active phenolic constituents, investigating their molecular targets, and evaluating the efficacy of the extracts in chronic treatment settings. This knowledge could support the development of *Acmella oleracea*-extract-based therapies as complementary strategies for managing chemotherapy-induced neuropathic pain and other similar debilitating conditions.

## Figures and Tables

**Table 1:** Amount of the bioactive molecules in the two dry extracts (DE) of AP and R used for the bioassays.

	HPLC-DAD			HPLC-MS
	mg/g DE			
Dry Extracts (DE)	Tot. Phenols	Tot. Alkylamides	Spilanthol	Spilanthol
AP	8.68 ^B^ ± 0.03	2.77 ^A^ ± 0.08	2.20 ^A^ ± 0.01	2.05 ^A^ ± 0.01
R	14.15 ^A^ ± 0.21	0.49 ^B^ ± 0.03	0.22 ^B^ ± 0.02	0.21 ^B^ ± 0.01

The two samples were constituted by a mixture of dried hydroalcoholic extract and maltodextrins in a ratio of 1:1 *w*/*w*. Data obtained by HPLC-DAD and by HPLC-MS are expressed as mean of triplicates (N = 3) ± SD. Different superscript letters indicate statistically significant differences between AP and R, as determined by one-way ANOVA followed by Tukey’s post hoc test (*p* < 0.0001).

**Table 2:** Absolute amounts of total phenols, spilanthol, and total alkylamides of AP and R dry extracts in administered doses during animal testing. Values are expressed as mg per kg of body weight (mg/kg bwt) and were calculated based on the concentrations (mg/g DE) reported in Table 1. Data represent the mean of triplicates (N = 3) ± SD.

Samples	Dose (mg DE/kg bwt)	mg/kg bwt		
		Total Phenols	Spilanthol	Total Alkylamides
AP	200	1.74 ± 0.01	0.44 ± 0.00	0.55 ± 0.02
	600	5.21 ± 0.02	1.32 ± 0.01	1.66 ± 0.05
	1200	10.42 ± 0.03	2.64 ± 0.01	3.32 ± 0.09
R	200	2.83 ± 0.04	0.04 ± 0.00	0.1 ± 0.01
	600	8.49 ± 0.13	0.13 ± 0.01	0.29 ± 0.02
	1200	16.99 ± 0.25	0.27 ± 0.02	0.58 ± 0.04

## Data Availability

The original contributions presented in this study are included in the article/Supplementary Materials. Further inquiries can be directed to the corresponding author.

## Supplementary Materials

The following supporting information can be downloaded at https://www.mdpi.com/article/10.3390/nu17162588/s1, Figure S1: ^1^H-NMR spectra.

## Abbreviations

The following abbreviations are used in this manuscript:

AP	Aerial parts
ATF3	Activating Transcription Factor 3
BDNF	Brain-derived neurotrophic factor
CMC	Carboxymethylcellulose sodium salt
Ire1α	Inositol-Requiring Enzyme 1 Alpha
LDH	Lactate dehydrogenase
MTT	3-(4,5-dimethylthiozol-2-yl)-2,5-diphenyltetrazolium bromide
Nf-H	Neurofilament heavy chain
OIPN	Oxaliplatin-induced peripheral neurotoxicity
R	Roots
SOD	Superoxide dismutase
