# Supplementary material for: Neuroprotective Potential of Acmella oleracea Aerial Parts and Root Extracts: The Role of Phenols and Alkylamides Against Neuropathic Pain
Source: Nutrients. 2025 Aug 8;17(16):2588. doi: 10.3390/nu17162588 (PMC12389297; doi:10.3390/nu17162588)
Supplement: Supplementary file 1 [file nutrients-17-02588-s001.zip › nutrients-3695164-supplementary.pdf]

Article

Neuroprotective Potential of *Acmella oleracea* Aerial Parts and Root Extracts: The Role of Phenols and Alkylamides Against Neuropathic Pain

Valentina Ferrara [1,†], Beatrice Zonfrillo [2,†], Maria Bellumori [2], Marzia Innocenti [2], Laura Micheli [1,*], Valentina Maggini [3], Daniel Venturi [1], Eugenia Gallo [3], Patrizia Bogani [4], Lorenzo Di Cesare Mannelli [1], Carla Ghelardini [1], Nadia Mulinacci [2] and Fabio Firenzuoli [3]

[1] Department of Neuroscience, Psychology, Drug Research and Child Health (NEUROFARBA), Section of Pharmacology and Toxicology, University of Florence, 50139 Florence, Italy; valentina.ferrara@unifi.it (V.F.); daniel.venturi1@unifi.it (D.V.); lorenzo.mannelli@unifi.it (L.D.C.M.); carla.ghelardini@unifi.it (C.G.)
[2] Department of Neuroscience, Psychology, Drug Research and Child Health (NEUROFARBA), Section of Pharmaceutical and Nutraceutical Sciences, University of Florence, via Ugo Schiff 6, 50019 Sesto Fiorentino, Italy; beatrice.zonfrillo@unifi.it (B.Z.); maria.bellumori@unifi.it (M.B.); marzia.innocenti@unifi.it (M.I.); nadia.mulinacci@unifi.it (N.M.)
[3] CERFIT, Research and Innovation Center in Phytotherapy and Integrated Medicine, Careggi University Hospital, 50134 Florence, Italy; eugenia.gallo@unifi.it (E.G.); fabio.firenzuoli@unifi.it (F.F.)
[4] Department of Biology, University of Florence, 50019 Sesto Fiorentino, Italy; patrizia.bogani@unifi.it
* Correspondence: laura.micheli@unifi.it; Tel.: +39-055-2758395

Supplementary Figure S1

a) Spilanthol

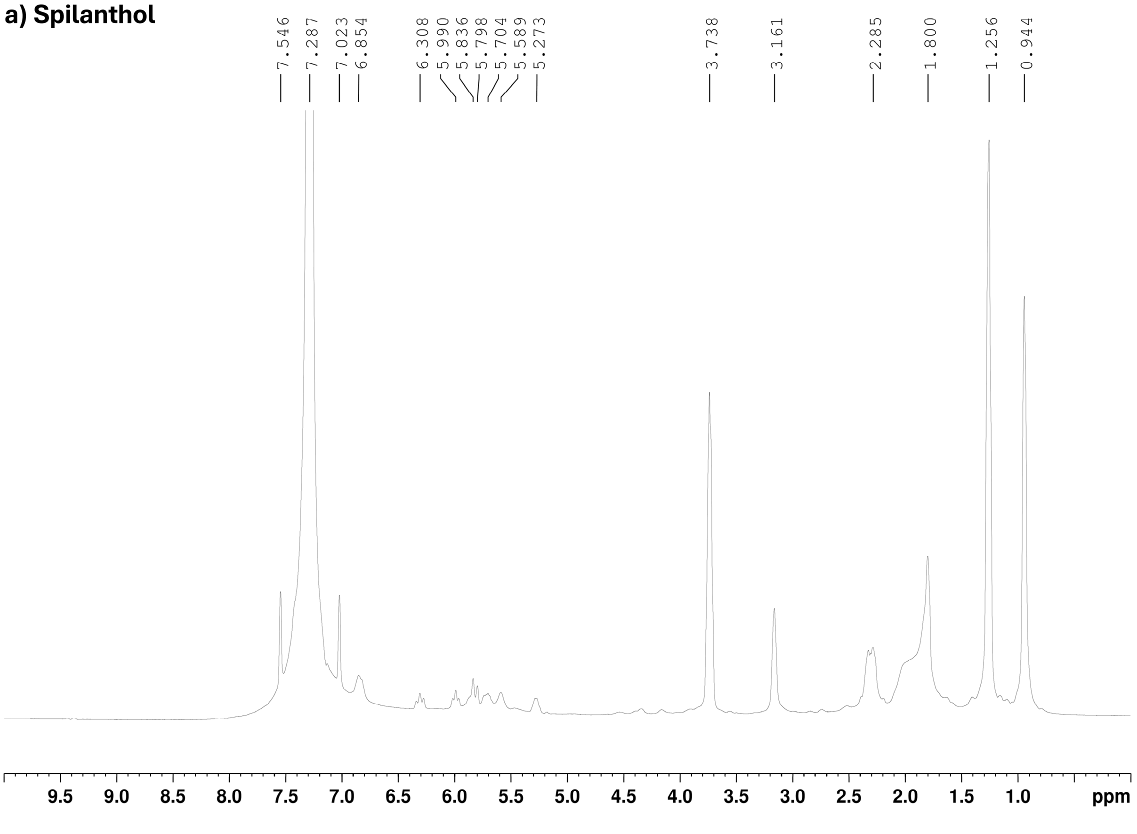

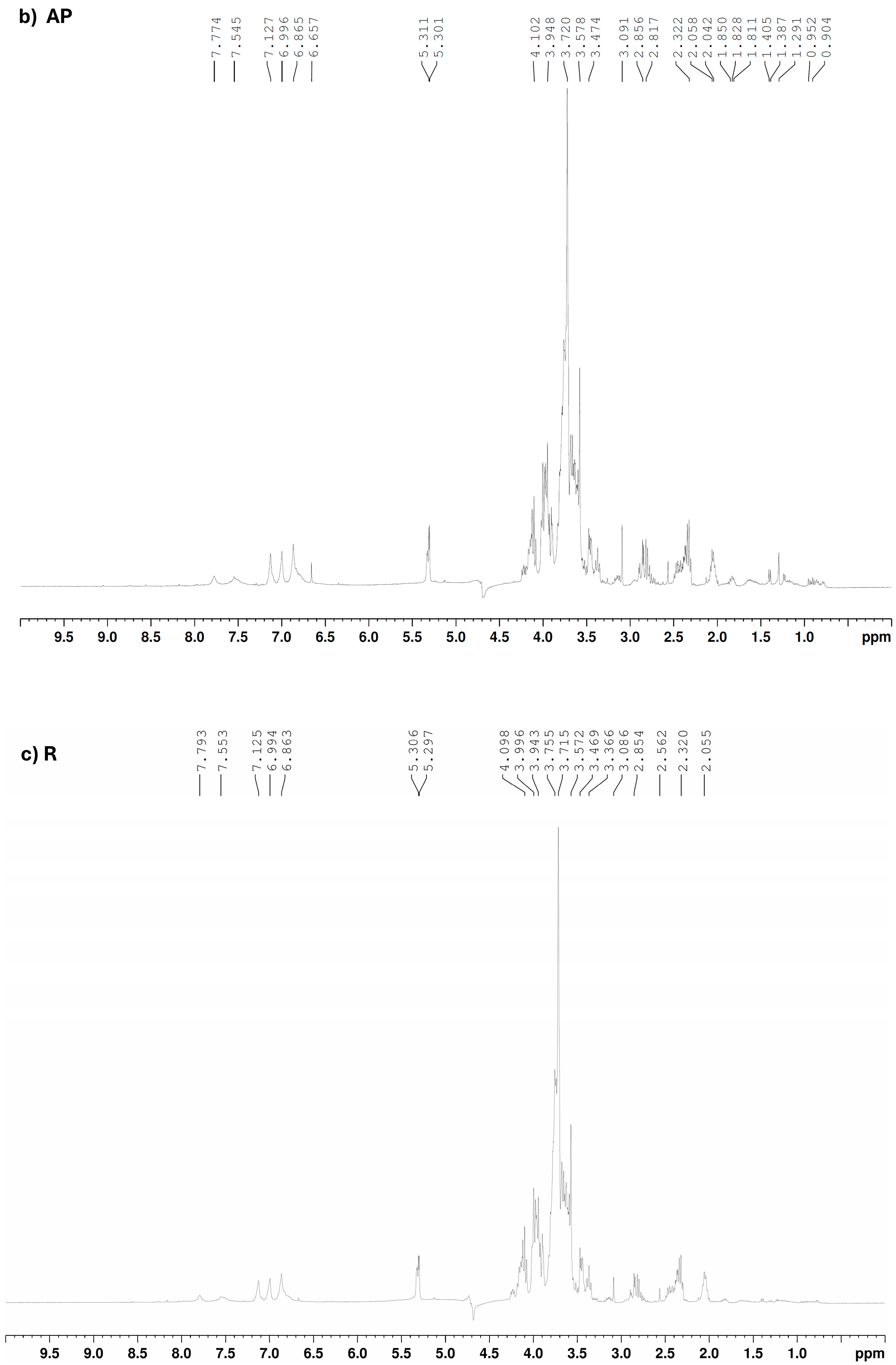

Supplementary Figure S1. ^{1}H-NMR spectra of a) spilanthol standard, b) aerial parts extract (AP), c) roots extract (R).
